# Retrospective Study on Liberation in Dogs Undergoing Mechanical Ventilation for Longer Than 24 Hours

**DOI:** 10.1111/vec.70090

**Published:** 2026-04-18

**Authors:** Abigail E. McNally, Thomas D. Greensmith, Stefano Cortellini

**Affiliations:** ^1^ Department of Clinical Science and Services The Royal Veterinary College, University of London North Mymms Hertfordshire UK

**Keywords:** positive pressure ventilation, spontaneous breathing trial, vasopressor, weaning

## Abstract

**Objective:**

To describe liberation in a population of dogs undergoing ≥24 h of mechanical ventilation (MV) and to assess for differences between successful and unsuccessful liberation attempts as well as adherence to criteria for veterinary and human readiness to discontinue MV.

**Design:**

Retrospective study.

**Setting:**

University teaching hospital.

**Animals:**

Forty‐seven client‐owned dogs undergoing ≥24 h of MV.

**Measurements and Main Results:**

Medical records were retrospectively reviewed; signalment, underlying disease, indications for MV, and survival to discharge were recorded. Data were collected up to 2 h before discontinuation of MV, noting ventilator settings and physiologic and blood gas variables. Twenty‐four of 47 (51.1%) cases undergoing MV were successfully liberated; of these, 22 (91.7%) dogs survived to discharge. Dogs were successfully liberated using various modes of ventilation, including assist control (15/24 [62.5%]), synchronized intermittent mandatory ventilation (8/24 [33.3%]), and pressure support ventilation (1/24 [4.2%]). Forty‐eight attempts at liberation occurred in 30 dogs. Of these, 24 of 48 (50%) were successful. Only end‐tidal carbon dioxide (ETCO_2_) was different between successful and unsuccessful liberation attempts, being higher in unsuccessful attempts (35 ± 6.8 vs. 43.7 ± 5.5 mm Hg; *p* < 0.0001). Sufficient information was available to assess adherence to veterinary liberation criteria in 31–36 liberation attempts, depending on the criterion. Liberation success did not differ between dogs that met the criteria and those that did not. Of dogs with complete data that were successfully liberated, 55% (10/18) did not meet current veterinary criteria to undergo a spontaneous breathing trial (SBT). The prevailing reason for failing to meet the veterinary criteria was cardiovascular instability (8/10), often due to vasopressor use (*n* = 7).

**Conclusions:**

Most successfully liberated cases did not fulfill the veterinary criteria for readiness to undergo an SBT. Dogs should not necessarily be precluded from undergoing an SBT or liberation attempt if they are receiving vasopressors but meet the remaining criteria.

**Abbreviations**
CPA: cardiopulmonary arrest
CPAP: continuous positive airway pressure
CRI: constant rate infusion
ETCO_2_
: end‐tidal carbon dioxide
IQR: interquartile range
MV: mechanical ventilation
PEEP: positive end‐expiratory pressure
PSV: pressure support ventilation
SBT: spontaneous breathing trial
SIMV: synchronized intermittent mandatory ventilation

## 1 Introduction

Over the last four decades, human literature has moved away from the term “weaning” to describe the discontinuation of mechanical ventilation (MV), instead embracing terms such as “liberation” or “discontinuation” to describe the complex challenges faced by patients and professionals during this multifaceted period of withdrawing support [1, 2]. It is widely reported in the human literature that the longer a patient remains dependent on MV, the more likely they are to have complications, complex liberation periods, and increased costs of care [3, 4, 5, 6]. Early identification of human patients suitable for liberation is therefore associated with improved clinical outcomes [7]. This has prompted the investigation of a series of liberation criteria that identifies patients suitable to begin the liberation process [8, 9, 10, 11, 12, 13, 14]. Numerous studies have allowed for the development of a stepwise approach to liberating human patients from MV, with many studies evaluating different protocols incorporated in clinical practice guidelines [15, 16, 17]. So great is the importance of liberation that alongside the multitude of indices and criteria present in human medical literature, many modern ICU ventilators offer semiautomated liberation systems. Through monitoring and real‐time intervention, these systems adapt to the needs of the mechanically ventilated patients and may shorten the overall duration of ventilation [18].

In people, potential candidates for liberation are recommended to undergo an initial spontaneous breathing trial (SBT) while intubated to ascertain their ability to sustain spontaneous breathing [9, 13]. In several randomized controlled trials in people, SBTs have been shown to reduce the time to liberation by 50%–66% compared with other methods, such as synchronized intermittent mandatory ventilation (SIMV) and pressure support ventilation (PSV) [19, 20, 21]. Daily assessments, including parameters such as PaO_2_:FiO_2_ ratio, ventilatory settings (such as positive end‐expiratory pressure [PEEP]), and subjective measures such as reduction in tracheobronchial secretions, are used to identify human patients suitable to undergo an SBT [22, 23]. Several indices that can be calculated during an SBT, among them the rapid shallow breathing index and dead space fraction (physiologic dead space to tidal volume ratio), may be used in conjunction with other parameters to indicate which patients are at increased risk of failing liberation [15, 24]. No veterinary literature to date has examined the use of such assessments and predictors in the discontinuation of MV, and no liberation guidelines currently exist for veterinary patients other than recommendations for SBT based on human criteria [25]. There are several differences in both the physiology and management of human and veterinary patients undergoing MV, making the direct translation of preliberation assessments and the appropriate timing for an SBT questionable. The suitability of current SBT recommendations in dogs is an area that has not yet been studied.

The primary aim of the current, single‐center study was to describe the existing practices regarding liberating dogs from MV and to review the dogs’ characteristics before and during successful and unsuccessful liberation attempts. The secondary aim of this study was to assess the suitability of current recommendations for veterinary SBTs and the selected human criteria [2, 26]. We hypothesized that dogs that met the current veterinary and human SBT guidelines would be more likely to be successfully liberated from MV than dogs that did not.

## 2 Materials and Methods

The electronic medical records of client‐owned dogs that underwent MV at the Queen Mother Hospital for Animals, Royal Veterinary College, between January 2016 and July 2022 were retrospectively reviewed. A search was performed for inclusion terminology, including “mechanical ventilation” or “ positive pressure ventilation,” and records were reviewed to ensure that patients underwent MV and met the inclusion criteria. Dogs that underwent continuous MV for ≥24 h were included in the study. Dogs ventilated for <24 h were excluded, as these were not considered to have undergone long‐term MV [27]. Dogs that lacked medical record information regarding ventilator settings and clinicopathologic data within 2 h of discontinuation of MV (either due to death or liberation) were also excluded.

Data collected included signalment, weight, underlying disease diagnosis, indication for MV, ventilation settings, physiologic and blood gas variables, presence of tracheostomy tube, liberation attempts, and survival to discharge. Dogs were classified as a brachycephalic breed if they were reported to have a brachycephalic conformation [28]. Dogs noted to be crossbreeds were reported as brachycephalic if their clinical record referred to a brachycephalic conformation.

Dogs were divided into two groups: those that were successfully liberated from MV defined as liberation from MV and spontaneously breathing for ≥24 h, and those that were not liberated. Those patients that were not liberated included those that failed liberation attempts (if performed) and underwent cardiopulmonary arrest (CPA) or euthanasia while still receiving MV. For dogs that were not liberated and underwent euthanasia or CPA during MV, data were recorded at the last recorded point during MV (within a maximum of 2 h). For patients that underwent multiple unsuccessful liberation attempts, the data closest to each attempt were collected within a maximum of 2 h before each attempt.

The underlying disease process of each dog was classified as neurologic, cardiac, pulmonary, or upper respiratory tract disease. Dogs were defined as having neurologic disease if their peripheral or central nervous system was affected, cardiac disease if ventilated during management for congestive heart failure or for marked hemodynamic instability from a primary cardiac disease process, respiratory disease if suffering from primary pulmonary parenchymal or pleural space pathology, and upper respiratory tract disease if suffering from an upper airway obstruction requiring MV. Resolution of underlying disease was determined based on clinical findings, laboratory data, and discontinuation of further therapy at the end of MV.

The indication for MV was recorded as either hypoxemia, hypoventilation, or mixed. Patients were categorized as hypoxemic if they had clinical signs of cyanosis, a PaO_2_ of <60 mm Hg, or, where blood gases were not available, an SpO_2_ of <90% despite supplemental oxygen [29]. Hypoventilation was defined as a PaCO_2_ of >55 mm Hg or a PvCO_2_ of >60 mm Hg or, where blood gas values were not available, as cases in which the clinical record indicated subjective clinician concern for hypoventilation, such as reduced spontaneous breathing efforts or apparent respiratory fatigue from prolonged respiratory distress [30]. The indication was defined as mixed if the dog exhibited clinical signs or blood gas values compatible with both hypoxemia and hypoventilation.

Due to variability in the timing of data recording, ventilation settings, physiologic parameters, and blood gas variables were collected as close as possible to MV liberation or death, with a maximum allowable time window of 2 h before the event. If there were multiple recordings within the 2‐h window, the data set closest to liberation was recorded. Where multiple liberation attempts were made, data were recorded within a 2‐h window before each attempt.

Ventilator‐related variables included ventilator mode, control variable, tidal volume, set respiratory rate, PEEP (both actual and set), peak inspiratory pressure, mean inspiratory pressure, set FiO_2_, and duration of MV. Clinical parameters included end‐tidal carbon dioxide (ETCO_2_), SpO_2_, and systolic blood pressure (as measured invasively or noninvasively). Noninvasive systolic blood pressures were either oscillometric or systolic Doppler measurements [31]. Blood pressure is measured at the institution following established guidelines [32, 33]. Where invasive blood pressure measurements were available, these data were recorded in preference to noninvasive blood pressure values [34].

Arterial or venous blood gas values^1^ were recorded, with arterial values used preferentially; if arterial values were not available, venous samples were used with the exclusion of PO_2_. Parameters recorded included blood pH, PaCO_2_ (mm Hg), PaO_2_ (mm Hg), and blood lactate (mmol/L). PaO_2_:FiO_2_ ratio was calculated using recorded PaO_2_ while in receipt of a known FiO_2_. If arterial blood gas values were unavailable, then the SpO_2_:FiO_2_ ratio was calculated. Recording of ETCO_2_ at the time of blood gas sampling is standard practice within our institution.

Liberation at our institution is not protocol driven, and approaches to liberation are clinician led. Animals were retrospectively defined as suitable for liberation if they met the criteria in Table 1. Human criteria included the Statlender and Singer and the Macintyre et al. criteria, which were selected to encompass both shorter and more comprehensive criteria [2, 26]. The number of liberation attempts for each dog was recorded in addition to whether or not the attempt was successful.

**TABLE 1:** Published criteria in the veterinary and human literature defining suitability for liberation from mechanical ventilation or a spontaneous breathing trial.

Veterinary guidelines [25]	Statlender and Singer criteria [26]	MacIntyre et al. criteria [2]
PaO_2_:FiO_2_/SpO_2_:FiO_2_ ≥200	PaO_2_:FiO_2_/SpO_2_:FiO_2_ ≥260	PaO_2_:FiO_2_/SpO_2_:FiO_2_ ≥150 PaO_2_ ≥60 mm Hg
PEEP ≤5 cm H_2_O	PEEP ≤5 cm H_2_O	PEEP ≤10 cm H_2_O
FiO_2_ ≤0.5	FiO_2_ ≤0.4	FiO_2_ ≤0.4
Cardiovascularly stable	pH ≥7.25	Cardiovascularly stable Afebrile (≤39.3°C) Absence of respiratory acidosis (pH ≥7.25) Electrolytes within normal limits

Abbreviation: PEEP, positive end‐expiratory pressure; SpO_2_, pulse oximetry value.

Animals were retrospectively considered to be cardiovascularly stable at liberation if they met the following criteria: systolic blood pressure >90 mm Hg without receipt of vasopressor agent(s), absence of tachycardia (defined as heart rate <160/min), and the absence of arrhythmias causing hemodynamic instability [14, 35, 36].

In dogs ventilated via orotracheal tube, a successful liberation attempt was defined as the ability to spontaneously breathe unaided for at least 24 h after extubation. Extubation was defined as removal of an endotracheal tube. In dogs ventilated via a temporary tracheostomy tube, a successful liberation attempt was defined as the ability to spontaneously breathe unaided for at least 24 h after disconnection from the ventilator regardless of the duration of time the temporary tracheostomy tube remained in place. Where patients had multiple liberation attempts, data as previously reported were collected for each attempt.

### 2.1 Statistical Analysis

Statistical analysis was performed using a commercial statistical package^2^. Continuous data were assessed for normality using the Shapiro–Wilks test. Normally distributed data sets were reported as mean ± SD and nonnormally distributed data sets as median and interquartile range (IQR). If the variable was not found to be normal across both groups (liberated and not liberated), data were considered nonnormal. Qualitative variables are reported as absolute and relative frequencies and proportions. Groups were compared using the Mann–Whitney *U*‐test for nonnormally distributed continuous variables or an unpaired *t*‐test for normally distributed continuous variables. For case counts ≤5, a *χ* ^2^ test or Fishers exact test was used to compare categorical data sets. Negative and positive predictive values and positive and negative likelihood ratios were calculated for the three criteria required to predict successful liberation. Statistical significance was set at *p* < 0.05.

## 3 Results

**FIGURE 1:**
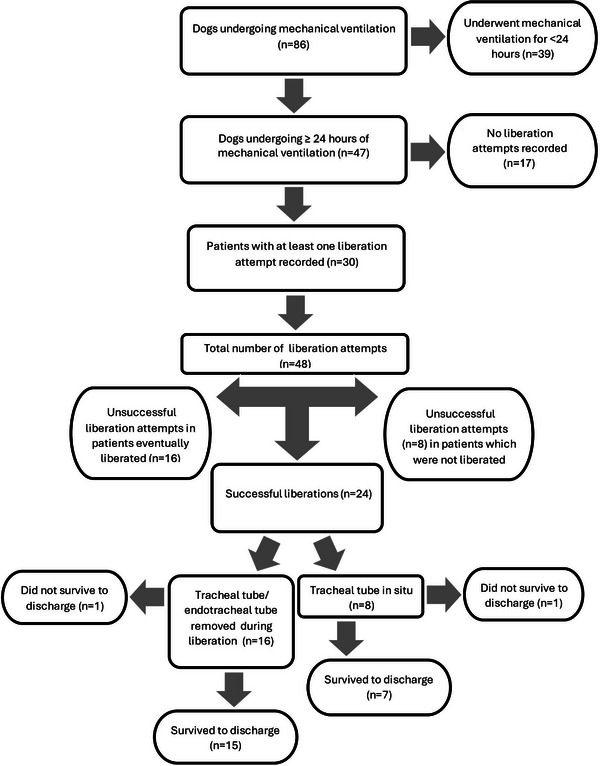
Flow chart detailing the study population of 47 dogs undergoing mechanical ventilation for ≥24 h.

Eighty‐six dogs were identified as having undergone MV between January 2016 and July 2022. Thirty‐nine dogs were excluded for MV <24 h (Figure 1), so 47 dogs were enrolled in the study.

Breeds included French Bulldog (*n* = 7 [14.9%]), crossbreed (*n* = 5 [10.6%]), Labrador Retriever (*n* = 3 [6.4%]), Golden Retriever (*n* = 3 [6.4%]), Cocker Spaniel (*n* = 3 [6.4%]), Dachshund (*n* = 3 [6.4%]), Bulldog (*n* = 2 [4.3%]), English Springer Spaniel (*n* = 2 [4.3%]), Shih Tzu (*n* = 2 [4.3%]), and one each (2.1%) of Greyhound, Lurcher, Lhasa Apso, Shetland Sheepdog, Beagle, Bichon Frise, Pug, Boston Terrier, Havanese, Pomeranian, Weimaraner, Boxer, Rottweiler, Yorkshire Terrier, Chow Chow, Vizsla, and Schnauzer.

Table 2 documents differences between the groups based on signalment, for which only age was significant, with liberated dogs being younger (median, 2 years, IQR: 0.6–5.2 years) than dogs that were not liberated (median, 7.7 years, IQR: 6.1–11 years) (*p* < 0.0001). There was no difference between the number of brachycephalic breeds in the liberated and not liberated groups (*p* = 0.92).

**TABLE 2:** Signalment, survival, underlying cause, and indication for mechanical ventilation in 24 dogs liberated and 23 dogs unable to be liberated from long‐term mechanical ventilation.

	Liberated (*n* = 24)	Not liberated (*n* = 23)	*p*‐value
Median age (IQR), years	2 (0.6–5.2)	7.7 (6.1–11)	0.0001
Weight, kg	14.25 (8.1–20.7)	9.9 (7.6–13.55)	0.2018
Sex			0.5265
Female intact	4/24 (16.7%)	2/23 (8.7%)	
Female neutered	7/24 (29.2%)	11/23(47.8%)	
Male intact	8/24 (33.3%)	5/23 (21.7%)	
Male neutered	5/24 (20.8%)	5/23 (21.7%)	
Brachycephalic conformation	7/24 (29.2%)	8/23 (34.7%)	0.92
Survival to discharge	22/24 (91.7%)	0/23 (0%)	N/A
Underlying disease process			0.47
Pulmonary (*n* = 28)	14/28 (50%)	14/28 (50%)	
Pneumonia (of undefined cause)	6/14 (42.8%)	7/14 (50%)	
Aspiration pneumonia	5/14 (35.7%)	7/14 (50%)	
Noncardiogenic pulmonary edema	2/14 (14.3%)	0	
Pleural space disease	1/14 (7.1%)	0	
Neurological (*n* = 10)	6/10 (60%)	4/10 (40%)	
Neuromuscular disease	3/6 (50%)	3/4 (75%)	
CNS disease	1/6 (16.7%)	1/4 (25%)	
Intervertebral disk disease	2/6 (33.3%)	0	
Upper respiratory tract obstruction (*n* = 7)	4/7(57.1%)	3/7 (42.9%)	
Laryngeal paralysis	1/4 (25%)	0	
Tracheal collapse	2/4 (50%)	0	
Brachycephalic obstructive airway syndrome	1/4 (25%)	3/3 (100%)	
Cardiac (*n* = 2)	0 (0%)	2/2 (100%)	
Post‐cardiopulmonary bypass	0	1/2 (50%)	
Congestive heart failure	0	1/2 (50%)	
Indication			0.52
Hypoxemia	9/24 (37.5%)	6/23 (26.1%)	
Hypoventilation	10/24 (41.7%)	9/23 (39.1%)	
Mixed	5/24 (20.8%)	8/23 (34.8%)	

Abbreviation: IQR, interquartile range.

Eight (33.3%) dogs from the liberated group had a temporary tracheostomy tube in place at the time of successful liberation from MV. Of these, four (50%) dogs had pulmonary pathology confirmed with imaging, including aspiration pneumonia (*n* = 3) and noncardiogenic pulmonary edema (*n* = 1). Three dogs had neurologic diseases, including C4–C5 myelopathy (*n* = 2) and lower motor neuron disease (*n* = 1). The remaining dog had a tracheostomy tube placed to manage upper respiratory tract obstruction due to a diphtheritic tracheal membrane, hypoplastic trachea, and dynamic airway collapse. Of these eight dogs, two were brachycephalic breeds.

The underlying disease processes of the two groups are shown in Table 2. No differences were identified between the liberated and not liberated groups (*p* = 0.47). The reasons for initiating MV in both groups are shown in Table 2. There was no difference identified between the groups (*p* = 0.52).

The ventilator settings and clinical parameters of the two groups are shown in Table 3. Subgroup analysis found the mode of MV used in liberated and not liberated dogs to be different (*p* = 0.00001), due to different proportions between groups that used assist control versus SIMV (*P* ≤ 0.001) and groups that used assist control versus pressure support ventilation (PSV) (*p* = 0.002). The proportion of patients managed with pressure versus volume control was higher in the not liberated group (*p* = 0.029), as were the prescribed respiratory rate (*p* = 0.004), FiO_2_ (*p* ≤ 0.001), PEEP (*p* ≤ 0.001), peak inspiratory pressure (*p* = 0.0031), and mean inspiratory pressure (*p* ≤ 0.001). The proportion of dogs that were cardiovascularly stable was not different between groups, nor was rectal temperature, PaCO_2_, or ETCO_2_. All other evaluated clinical parameters were worse in the group that was not liberated from MV (Table 3).

**TABLE 3:** Differences in ventilator settings and clinical parameters at a time point within 2 h of successful liberation or death in 47 dogs undergoing mechanical ventilation for ≥24 h.

Variable	Liberated (*n* = 24)	Not liberated (*n* = 23)	*p*‐value
Ventilator settings			
Mean duration of ventilation (h)	54 (37.6–71)	65.7 (36.5–78.8)	
Mode of ventilation			0.00001
Assist control	15 (62.5%)	20 (86.9%)	
Synchronized intermittent mandatory ventilation	8 (33.3%)	1 (4.3%)	
Pressure support ventilation	1 (4.2%)	2 (9.7%)	
Control variable			0.029
Volume	12 (50%)	4 (17.4%)	
Pressure	12 (50%)	19 (82.6%)	
Set respiratory rate (/min)	28 (20–37.5)	42 (36–50)	0.0004
Fraction of inspired oxygen (%)	35 (30–40)	80 (60–100)	0.0001
Tidal volume (mL/kg)	8.51 (7.8–9.2)	7.94 (6.6–9.9)	0.35
Positive end‐expiratory pressure (cm H_2_O)	3 (2.9–4.6)	5 (4.5–6.3)	0.0002
Peak inspiratory pressure (cm H_2_O)	13.3 ± 5	19.6 ± 7.2	0.0031
Mean inspiratory pressure (cm H_2_O)	6 ± 1.8	10.3 ± 4.2	0.0001
Clinical parameters			
Cardiovascularly stable	10 (*n* = 19)	6 (*n* = 18)	0.33
Respiratory rate (/min)	31 (23.5–40.75)	48 (40–64)	0.0006
Heart rate (/min)	87.6 ± 37.8	127.8 ± 23.2	0.0006
Systolic blood pressure (mm Hg)	136.9 ± 27.2	109.8 ± 42.6	0.039
Temperature (°C)	38.5 ± 0.8	38.3 ± 1.2	0.66
SpO_2_ (%)	95 (92.5–96)	89.5 (82.8–92.3)	< 0.0001
SpO_2_:FiO_2_	274 (237.5–318.3)	104.1 (84.5–149.3)	< 0.0001
PaO_2_ (mm Hg)	115.4 ± 40.9	88.9 ± 27.3	0.045
PaO_2_:FiO_2_	346.25 (257.6–432)	107.7 (79.3–185.8)	< 0.0001
PaCO_2_ (mm Hg)	44.5 ± 11.6	51.2 ± 15.5	0.31
PvCO_2_ (mm Hg)	46.8 (41.7–56.1)	69.6 (56.2–97.5)	0.094
End‐tidal carbon dioxide (mm Hg)	35 ± 6.8	39.2 ± 11.7	0.15
pH	7.339 ± 0.1 (*n* = 17)	7.173 ± 0.16 (*n* = 16)	0.0012
Lactate (mmol/L)	0.6 (0.3–6) (*n* = 18)	1.1 (0.4–9.2) (*n* = 16)	0.004

*Note*: For variables in which not all patients had available information, the number (*n*) of cases with this information is listed.

Abbreviation: SpO_2_, pulse oximetry value.

**FIGURE 2:**
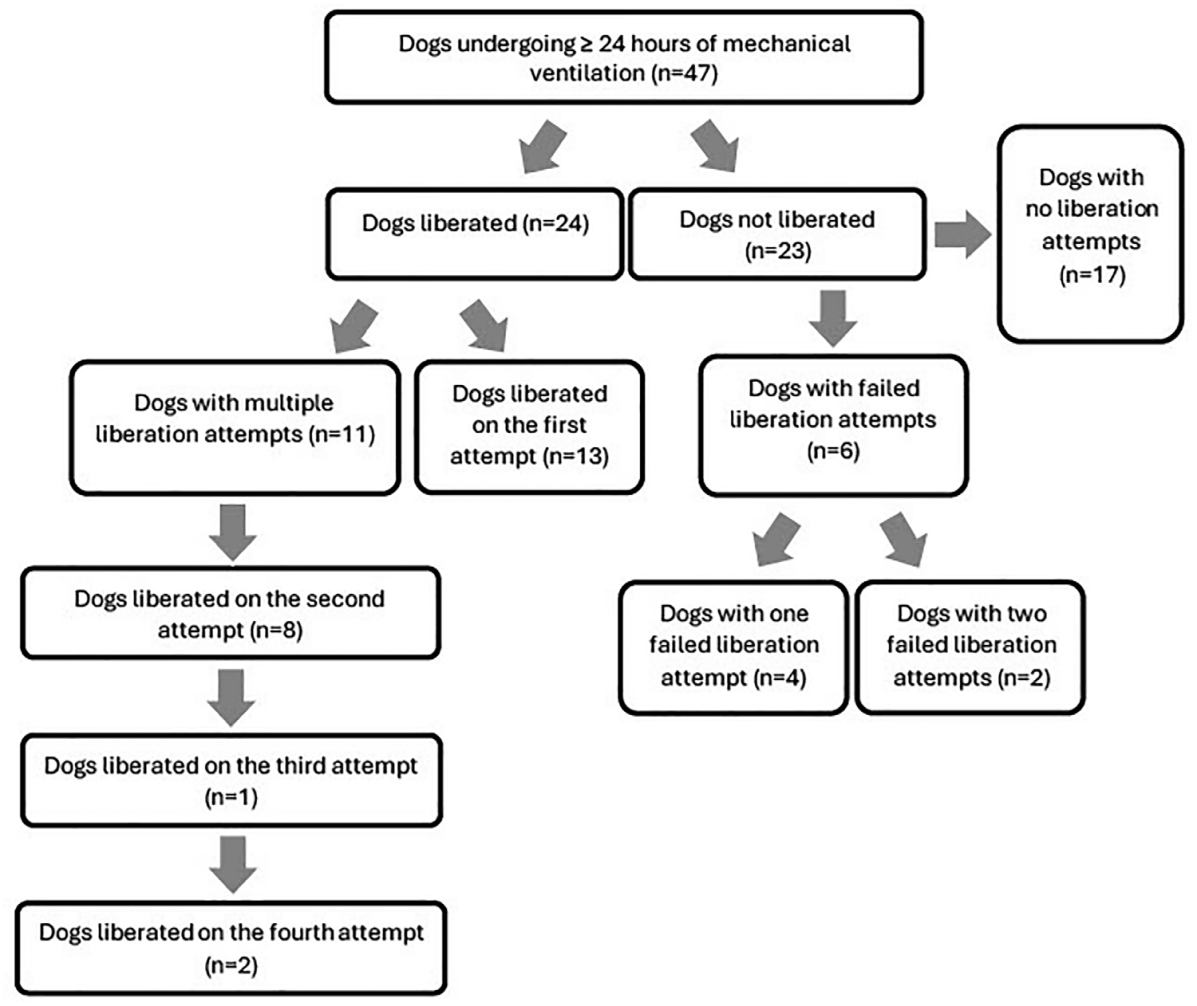
Details of mechanical ventilation liberation attempts in 47 dogs undergoing mechanical ventilation for ≥24 h.

A total of 48 liberation attempts were identified in 30 dogs. Of these liberation attempts, 24 (50%) were successful, and most (13/24) dogs were successfully liberated on first attempt. The average duration of a liberation attempt was 1.35 h (0.5–3 h). During successful liberation attempts, dogs with available information (21/24) were most frequently receiving three constant rate infusions (CRIs) (13/21). Four of 21 dogs were receiving four CRIs, and three were receiving two. Only one dog, which had a tracheostomy tube in place and was conscious throughout ventilation, received no sedative CRIs but was managed with a single bolus of 5 µg/kg acepromazine during the liberation process. The most frequently administered CRIs were propofol (20/21) and fentanyl (18/21). A breakdown of details of the sedative medications and ventilator settings is shown in Table S1. Figure 2 shows the breakdown of liberation attempts for each group.

A comparison of ventilator settings and clinical parameters between successful and failed liberation attempts is shown in Table 4. Ventilator data were not available for two of the failed liberation attempts; therefore, 46 attempts were included in the further analysis between successful and failed liberation groups. Insufficient data were available for a number of measures, most notably blood–gas analysis, precluding the calculation of PaO_2_:FiO_2_ ratios. For some parameters, data were not available in all cases; where applicable, the number of cases is provided. Only ETCO_2_ was different between successful and failed liberation attempts (*p* < 0.001).

**TABLE 4:** Differences in ventilator settings and clinical parameters between successful and failed liberation attempts made in 30 dogs undergoing long‐term mechanical ventilation.

	Successful liberation attempts (*n* = 24)	Failed liberation attempts (*n* = 22)	*p*‐value
Ventilator settings			
Mode of ventilation			0.00005
Assist control	4/24 (16.6%)	18/22 (81.8%)	
Synchronized intermittent mandatory ventilation	13/24 (54.2%)	2/22 (9.1%)	
Pressure support ventilation	7/24 (29.2%)	2/22 (9.1%)	
Control variable			> 0.99
Volume	11/22 (45.8%)	10/22 (45.5%)	
Pressure	11/22 (45.8%)	12/22 (54.5%)	
Not recorded	2/24 (8.3%)		
Set respiratory rate (/min)	28 (20–37.5) (*n* = 24)	25.5 (20–32) (*n* = 22)	0.11
Fraction of inspired oxygen (%)	35 (30–40) (*n* = 24)	30 (30–40) (*n* = 22)	0.69
Tidal volume (mL/kg)	8.51 (7.8–9.2) (*n* = 24)	8.36 (7.5–9.3) (*n* = 22)	0.42
Positive end‐expiratory pressure (cm H_2_O)	3 (2.9–4.6) (*n* = 24)	4 (3–5) (*n* = 22)	0.29
Peak inspiratory pressure (cm H_2_O)	13.3 ± 5 (*n* = 24)	13.7 ± 5.5 (*n* = 22)	0.79
Mean inspiratory pressure (cm H_2_O)	6 ± 1.8 (*n* = 24)	6.7 ± 2.6 (*n* = 21)	0.28
Clinical parameters			
Heart rate (/min)	81 (56.3–112.5) (*n* = 18)	76 (63–104.5) (*n* = 15)	0.74
Respiratory rate (/min)	34 ± 14.2 (*n* = 24)	29 ± 7.8 (*n* = 22)	0.14
Temperature (°C)	38.5 ± 0.8 (*n* = 17)	38.3 ± 0.6 (*n* = 15)	0.54
Systolic blood pressure (mm Hg)	136.9 ± 27.2 (*n* = 18)	124.3 ± 36.6 (*n* = 15)	0.27
SpO_2_ (%)	95 (92.5–96) (*n* = 23)	95 (93–96) (*n* = 21)	0.78
SpO_2_:FiO_2_	274 (237.5–318.3) (*n* = 23)	300 (235–326.7) (*n* = 21)	0.41
End‐tidal carbon dioxide (mm Hg)	35 ± 6.8 (*n* = 24)	43.7 ± 5.5 (*n* = 22)	< 0.0001

Abbreviation: SpO_2_, pulse oximetry value.

Of the 46 liberation attempts performed for which contemporaneous ventilator data were available, 12 lacked sufficient patient data to be evaluated against veterinary liberation criteria, yielding 34 liberation attempts in 21 dogs that could be evaluated. Eleven of the 46 liberation attempts did not have a blood pressure available, and one attempt did not have an SpO_2_ measurement recorded. When successful liberation attempts were reviewed, eight of 24 met the veterinary criteria, while 10 of 24 did not. Of the unsuccessful liberation attempts, six of 22 met the criteria, while 10 of 22 did not. Of the 10 successful liberation attempts that did not meet the criteria, eight were identified as not cardiovascularly stable. In seven of these eight attempts, patients were receiving noradrenaline infusion at the time of liberation from MV, and one was tachycardic with a heart rate of 176/min. Of the patients receiving noradrenaline at the time of a successful liberation attempt, none were defined as hypotensive, with an average systolic blood pressure where available (6/7) of 120 mm Hg. All patients receiving noradrenaline at the time of liberation were receiving a dose between 0.1 and 0.3 µg/kg/min. The other two liberation attempts failed to meet the veterinary criteria due to a PaO_2_:FiO_2_ ratio of 127 and a PEEP of >5 cm H_2_O, respectively.

Of the 10 unsuccessful liberation attempts that failed to meet the criteria, eight were identified as not cardiovascularly stable. In four of these eight attempts, patients were receiving noradrenaline infusion at the time of the liberation attempt; none of these dogs were defined as hypotensive, with an average systolic blood pressure of 127 mm Hg. Of the remaining cases, three were hypotensive with an average systolic blood pressure of 76 mm Hg, while one had an unstable arrythmia reported. Of the remaining liberation attempts, one of two failed to meet the veterinary criteria due to receiving a PEEP of 6.5 cm H_2_O, while the other was receiving an FiO_2_ ≥0.5. There was no difference in liberation attempt success between dogs that met the criteria and those that did not (*p* = 0.74).

### 3.1 Statlender and Singer Criteria

Of the 46 liberation attempts performed, 10 lacked sufficient data for comparison to the Statlender and Singer criteria. Data missing at the time of liberation included blood gas analysis in three of 46, blood pressure or heart rate in six, and SpO_2_ in one dog. When reviewing successful liberation attempts, 12 dogs met the Statlender and Singer criteria, while six did not. Of the unsuccessful liberation attempts, 11 met the criteria, while seven did not. Of the six successful liberation attempts not meeting these human criteria, three (3/6 [50%]) patients had a PaO_2_:FiO_2_ ratio <260, one (1/6 [16.7%]) had a PEEP ≥5 cm H_2_O, and two (2/6 [33.3%]) had a respiratory acidosis with a pH <7.25. Of the unsuccessful liberation attempts that failed to meet these criteria, four of seven had a PaO_2_:FiO_2_ ratio <260, and one had a significant respiratory acidosis with a pH <7.25. Both of the remaining patients were receiving a PEEP ≥5 cm H_2_O. With respect to liberation attempt success, there were no differences between dogs that met the criteria and those that did not (*p* > 0.99).

### 3.2 MacIntyre et al. Criteria

Of the 46 liberation attempts, 15 lacked sufficient data for evaluation by the MacIntyre et al. criteria. Eleven of 46 liberation attempts did not have a blood pressure or a heart rate recorded, one did not have a PaO_2_ or an SpO_2_, and three did not have an available blood gas analysis. When successful liberation attempts were reviewed, six met the criteria, while 12 did not. Of the unsuccessful liberation attempts, five met the criteria, while eight did not. Of the 12 liberated patients not meeting the MacIntyre et al. liberation criteria, six (6/12 [50%]) failed due to cardiovascular instability alone, and two (16.6%) failed due to cardiovascular instability and respiratory acidosis. Three (25%) cases were febrile, while the final case was receiving FiO_2_ ≥0.5. Eight unsuccessful liberation attempts did not meet the criteria. Of these, seven were classified as cardiovascularly stable. Three dogs were receiving noradrenaline, while three were hypotensive, and the final case exhibited unstable cardiac arrythmias at the time of attempted liberation. When liberation attempts were compared, there was no difference between dogs that met the human criteria and those that did not (*p* > 0.99). Positive and negative predictive values and likelihood ratios for the liberation criteria are displayed in Table 5.

**TABLE 5:** Adherence to liberation criteria, success or failure of liberation, and related positive predictive value, negative predictive value, positive likelihood ratio, and negative likelihood ratio in 46 attempts to liberate 30 dogs from mechanical ventilation.

	Veterinary criteria (*n* = 34 liberation attempts evaluated)		Statlender and Singer criteria (*n* = 36)		MacIntyre et al. criteria (*n* = 31)	
	Met criteria	Did not meet criteria	Met criteria	Did not meet criteria	Met criteria	Did not meet criteria
Unsuccessful liberation attempt	6	10	11	7	5	8
Successful liberation attempt	8	10	12	6	6	12
Positive predictive value	57.1% (95% CI: 37.1–75.1)		52.2% (95% CI: 40–64.1)		54.6% (95% CI: 31.7–75.6)	
Negative predictive value	50% (95% CI: 36.3–63.7)		53.9% (95% CI: 32.8–73.6)		40% (95% CI: 28–53.4)	
Positive likelihood ratio	1.19 (95% CI: 0.52–2.68)		1.09 (95% CI: 0.67–1.79)		0.87 (95% CI: 0.34–2.24)	
Negative likelihood ratio	0.89 (95% CI: 0.51–1.56)		0.86 (95% CI: 0.4–2.1)		1.08 (95% CI: 0.63–1.86)	

Abbreviation: CI, confidence interval.

Twenty‐three dogs (23/47 [48.9%]) were not liberated from MV and thus did not survive to discharge. Seventeen of these had no liberation attempt, of which 12 were euthanized and five suffered CPA. Nine of 12 (75%) cases were euthanized because of a perceived poor prognosis by the clinician, and the remaining three (25%) cases had no reason for euthanasia recorded.

Twenty‐four of 47 (51.1%) dogs were liberated from MV. Of the dogs that had undergone MV for hypoxemia, nine of 15 (60%) were successfully liberated and eight (53.3%) survived to discharge. Of the dogs that underwent MV for hypoventilation, 10 of 19 (52.6%) cases were successfully liberated and nine (47.4%) survived to discharge. Five of 13 (38.5%) cases ventilated for a mixed disorder were liberated and survived to discharge. Two cases that were liberated did not survive to discharge: one suffered a repeat aspiration event, and prognosis was perceived as poor in addition to financial constraints. The other dog remained tetraparetic due to a C4–C5 spinal segment lesion and was euthanized due to a perceived poor prognosis. Overall, 22 of 47 (46.8%) patients survived to discharge.

## 4 Discussion

The current retrospective study describes a single‐center population of dogs that underwent ≥24 h of MV and associated liberation attempts. In addition, differences between successful and unsuccessful liberation attempts are characterized, which will help inform prospective work. Survival to discharge in the current study is higher (46.7%) than other studies (23.4%–29%), although both the underlying disease processes and indications for MV are broadly similar [27, 37]. The reason for this difference is not readily apparent, although successful liberation followed by death was uncommon (occurring in only two patients) in this study. It is important to note that our sample size was relatively small in comparison with other studies, which limits the strength of further analysis. Regarding underlying disease processes, no dog ventilated for primary cardiac disease was liberated from MV, despite previous reports of survival as high as 77% [38]. However, the small number of dogs ventilated for cardiac disease in this study (2/47) and the uncommon indication for one of these patients (following open surgical mitral valve repair) may explain this discrepancy. Interestingly, there was no difference in the proportion of brachycephalic dogs between the liberated and not liberated groups, and temporary tracheostomy tubes were most commonly used in patients that were not brachycephalic (6/8), which may reflect their use in the ventilation of patients with neuromuscular diseases. Whenever possible, patients with polyradiculoneuritis or tetanus are allowed to remain conscious while receiving MV at the authors’ institution, both to lower the cost of treatment and to reduce complications by maintaining a functional epiglottis. Future studies with larger sample sizes could help to evaluate the effectiveness of this ventilatory strategy compared with alternative approaches.

As expected, there were numerous differences between the population of dogs that were liberated and those that died or were euthanized while receiving MV, many of which reflect the time period of data collection. For example, values in the not liberated group were as close as possible to death, up to a maximum of 2 h prior; therefore, data such as oxygenation indices, ventilator settings, and clinicopathologic variables would be expected to be poor. One notable difference that is less readily explainable is the difference in age, with a median of 2 versus 7.7 years (*p* = 0.0001) in dogs liberated from MV and dogs that were not, respectively, despite having no difference in underlying disease processes or reason for initiation of MV. Older age in people has been associated with decreased chances of liberation, and similar data exist in veterinary medicine based on several previous veterinary studies [27, 39, 40], in which each additional year of age was associated with a 10% reduced rate of survival to discharge [27].

The overall number of dogs liberated in the current study (24/47 [51.1%]) is difficult to interpret in relation to other veterinary studies, given the differences in inclusion criteria, data collected, and disease severity. Several studies in dogs report overall liberation between 27% and 47%; however, a direct comparison cannot be made as these included dogs ventilated for <24 h [37, 41]. In studies of dogs ventilated for >24 h, liberation success has been reported as 43% and 44% [27, 37]. By selecting a patient population ventilated for ≥24 h, it is likely that several distinct populations of dogs that undergo short‐term MV were missing from the current study population. Such populations include both less severely affected dogs, such as those requiring short‐term support, as well as dogs that may be ventilated for short periods while awaiting family‐witnessed euthanasia and those with rapidly progressive, severe disease refractory to treatment. The latter population is unlikely to have been suitable to undergo liberation attempts, and those successfully liberated in <24 h are unlikely to have needed a true liberation process [25], rendering them less relevant to the current study. As is common in veterinary studies, euthanasia is another factor that may affect the study population. More than half of the unliberated population (12/23 [52.2%]) was euthanized before liberation attempt, which affects the estimation of successful liberation. The conclusions drawn from this study are limited by its small sample size. The broader population of dogs requiring MV may not be accurately reflected, potentially affecting the generalizability of the results.

In the current study, there were no differences noted in successful or unsuccessful liberation attempts depending on the mode of ventilation used (*p* = 0.13). Relatively little supporting evidence exists for the most commonly applied liberation techniques in veterinary medicine, and it varies markedly between previous studies [27, 37]. Several different modes of ventilation were used in this study, likely reflective of a variety of liberation techniques and clinician preference. The retrospective nature of this study makes it impossible to discern reasons the clinicians chose a specific ventilation mode or liberation strategy. Of all successful liberation attempts in this study, 62.5% used assist control ventilation until liberation. This suggests patients had MV abruptly withdrawn for a period while monitoring their ability to maintain their own respiratory workload, most reflective of an SBT. Although various protocols exist in the human literature, an SBT generally lasts 30–120 min and consists of pausing mandatory ventilation while observing respiratory and cardiovascular parameters (e.g., respiratory rate and rhythm, heart rate, blood pressure) and assessing the adequacy of pulmonary and ventilatory function (e.g., blood gas analysis) [4]. In people, each SBT is scored against objective and subjective indices to indicate success or failure [42]. Research in people suggests that no single parameter can predict if an SBT can be safely attempted [3, 15], and inappropriately timed SBTs can lead to increased respiratory muscle fatigue and hemodynamic instability [21].

The proportion of dogs in which SIMV was used in the current study during liberation was 33%, which is similar to the 22% in a recent report [37] and in contrast to an older study that documented that SIMV was the predominant liberation mode used in 49% of cats and dogs [27]. Typically, when using SIMV, ventilatory support is gradually withdrawn during liberation, allowing the animal to gradually resume the work of breathing through supported spontaneous breaths. In people being liberated from MV, SIMV has fallen out of favor, being cited as the preferred method by clinicians in 90.2% of cases in the 1980s [43] but now favored by only 0%–6% [44]. This decline is likely due to evidence documenting worsening respiratory muscle fatigue and a lower rate of successful liberation when using SIMV compared with other methods, such as a daily SBT [20].

The final mode of ventilation identified in the current study was PSV, which was used in a single successful liberation attempt. During PSV, a low level of pressure support to spontaneous breaths helps to overcome the increased resistance to breathing caused by an indwelling endotracheal tube, aiming to provide work of breathing as close to normal as would occur if the patient were extubated [45]. In people, however, some authors suggest this may provide a false sense of security and that a period of time breathing independently without any support can help to ensure the patient is truly ready for the discontinuation of MV and extubation [46, 47, 48, 49]. The use of continuous positive airway pressure (CPAP) was not reported in this study despite a recent veterinary study reporting it being the most common method of liberation, with CPAP being used in 66% of cases [37]. The current study considers the liberation practices of a single institution, with a single dog transitioned to PSV and none transitioned to CPAP. Many factors, including clinician experience and preference, as well as patient‐oriented factors and the availability of equipment, determine the mode of MV used during liberation. The modes of liberation used in the current study may therefore not be reflective of other institutions, and further research is required to assess the success of modes such as PSV and CPAP.

There are difficulties in the application of liberation predictors to veterinary medicine, as our patients have a wider range of respiratory rates, weights, and tidal volumes than those typically seen in human medicine [25]. Adjunct to this, to tolerate MV, veterinary patients are predominantly under a greater degree of sedation or anesthesia, resulting in a reduced ability to eliminate respiratory secretions and cough, among other challenges similar to those faced by a pediatric human population. This study documented that many patients not meeting liberation suitability criteria can still be successfully liberated from MV. Of the 18 successful liberation attempts, the veterinary criteria were met in only eight instances, or in six instances for the MacIntyre et al. criteria. Using the Statlender and Singer criteria, a greater number (12/18) of these cases would have met the criteria, but the number of dogs that could be successfully liberated from MV would still be underestimated [2, 26]. None of the assessed criteria distinguished between successful and unsuccessful liberation attempts; although given the small study population, there could be a type 2 error; both of the positive and negative predictive values and the likelihood ratios suggest that the three criteria should be interpreted with caution as pertains to MV liberation in dogs. The negative predictive values and negative likelihood ratio results, especially for the veterinary criteria, may need to be interpreted with caution, as the same criteria could have been used in forming clinician judgment to trigger the liberation process, limiting the chances of initiating the process in a patient not fulfilling those same criteria. This study retrospectively applied human criteria for liberation from MV, but the authors recognize that these two sets of criteria address only a small fraction of the comprehensive and multifaceted approaches present in the human literature.

The most common reason for dogs not to meet the liberation criteria was the recommendation for cardiovascular stability before an SBT. Although the current veterinary recommendation cites cardiovascular stability, there are no specific recommended criteria; however, specific recommendations in people do exist and include the absence of vasopressor agent use, hence its inclusion in this study definition of cardiovascular stability. The majority (8/10 [80%]) of the liberation attempts that were successful despite not meeting the veterinary criteria were due to the patient not being considered cardiovascularly stable, which in almost all cases (7/8 [87.5%]) was because vasopressors (noradrenaline) were still being received. Of the dogs successfully liberated while receiving vasopressors in this study, all had systolic blood pressure >90 mm Hg; therefore, if vasopressor support were to be removed from the definition, 15 of 18 (83.3%) of the successfully liberated cases would have met veterinary criteria. A prospective study in people has identified no increased risk of repeat intubation in patients liberated from MV while receiving low‐dose vasopressors [50], suggesting that dogs requiring ongoing low‐dose vasopressor use should not necessarily be precluded from undergoing SBTs or liberation attempts when remaining criteria suggest they could be liberated.

In veterinary medicine, SBTs are routinely performed at the point where an animal is thought to be ready to be liberated from MV [25]. This is in contrast to human patients, in which SBTs are often performed daily in approximately two thirds of patients as part of a global assessment of readiness to liberate [51]. In the human literature, only 13% of patients extubated after a successful SBT required repeat intubation compared with approximately 40% of dogs in which an SBT was not performed and only checklist criteria were used [3, 20, 52]. Evidence from human multicenter prospective studies has identified that an increased interval between the first point of eligibility for liberation and the first liberation attempt is associated with increased liberation failure [51]. This differing approach may result in a population of animals that could feasibly be liberated from MV but in which an SBT is not attempted; thus, a daily SBT could be beneficial for the early identification of veterinary patients that could be liberated from MV. The use of daily SBTs has become increasingly frequent at our study center, even in cases where liberation success might not be expected. Prompt liberation from ventilation may reduce the costs of ongoing care as well as the complications and physiologic impacts of long‐term MV, as is reported in the human literature [23].

Of those patients successfully liberated, 13 of 24 (54.2%) were liberated on their first attempt, with eight (33.3%) being liberated on their second attempt and the remainder requiring multiple attempts. Due to the retrospective nature of this study, it is not possible to interpret why almost half (11/24 [45.8%]) the dogs failed primary liberation attempts, as, in many cases, no specific reason for failure was found in the medical record. At the study institution, all mechanically ventilated patients are strictly barrier nursed, which precludes ready notation in the paper medical record unless a specified scribe is available, due to the risk of contamination of the patient's environment. As liberation attempts may last several hours, failed attempts (including patients that are extubated and subsequently reintubated) require clinicians to recollect and record data, which appears to be lacking in the current study and represents a marked limitation. Prospective data collection in a standardized way or the use of multi‐device electronic patient records (such as the simultaneous capture of multiparameter monitor values, ventilator parameters, and other patient care devices into a single data stream) would help mitigate this problem.

It must also be considered that unsuccessful attempts are an expected part of liberation from MV. A patient initially failing to discontinue support should not be considered as a definitive setback to achieve ventilator‐free status but rather as a step within the liberation process that might require several attempts and extend over multiple days. Patients in this study that failed the initial liberation attempt may have undergone a process resembling an SBT; however, due to the constraints of a retrospective study, accurately capturing this information is challenging. In human literature, around one third of patients reportedly fail the initial SBT [53]. A study of human patients with acute respiratory distress syndrome who failed SBTs found that 13 of 17 (76%) developed hypercapnia as a result of worsening pulmonary mechanics and a rapid shallow breathing pattern [54]. Among the criteria indicating patients are intolerant of an SBT, human guidelines suggest an increase in PaCO_2_ of >10 mm Hg and that the liberation attempt should be abandoned [55]. Various human studies have integrated both ETCO_2_ and PaCO_2_ into more sophisticated analysis of liberation, but none have been rigorously tested or found sufficient to successfully predict liberation alone [56, 57]. Veterinary guidelines similarly suggest an ETCO_2_ of >50 mm Hg among the criteria indicating a patient has failed an SBT [25]. Identifying patients poorly tolerant of SBT is vital, as an unsuccessful SBT of just 10 min in people can result in significant left ventricular dysfunction and cardiovascular stress [57]. Further prospective studies are needed.

There are many limitations to the current retrospective study, such as the inability to infer causality, as well as the variability in detail within the medical record and confounding effects of clinician preference and discretion in clinical decision‐making, which were variably recorded. As is common in veterinary studies, euthanasia and financial factors also likely altered the study population and its management in ways that can be difficult to predict. The definition of cardiovascular stability was derived from human guidelines and may not be applicable to veterinary patients.

In conclusion, the human literature suggests daily SBTs are the most frequently used method to evaluate a patient's readiness to discontinue MV [5, 51]. Despite this, there has been no evaluation of SBTs in the veterinary literature, indicating a lack of data surrounding the appropriate terminology, timing, approach, duration, suitability, and outcome. The current study describes a population of dogs liberated from MV and highlights that many successful liberations did not meet the current criteria found in either the veterinary or human literature. Although these criteria were applied retrospectively and only cover a fraction of the extensive human practices, an initial insight is provided into the need for further prospective research in veterinary‐specific MV liberation practices, including predictors and analysis of specific subpopulations of ventilated patients. The study also emphasizes that dogs receiving vasopressors should not necessarily be precluded from liberation attempts or SBTs. Future research should consider the implementation of updated terminology, proposed structure for liberation attempts, and evaluations of SBT in the veterinary population.

## Author Contributions

**Abigail E. McNally**: data curation, formal analysis, writing – original draft. **Thomas D. Greensmith**: formal analysis, supervision, writing – review and editing. **Stefano Cortellini**: formal analysis, supervision, writing – review and editing.

## Conflicts of Interest

The authors declare no conflicts of interest.

## Supporting information




**Supporting File 1**: vec70090‐sup‐0001‐SupMat.docx.

## Endnotes

1 ABL90 FLEX blood gas analyzer, Radiometer Limited, England, UK.

2 GraphPad Prism 10.0.3 for Windows, GraphPad Software, San Diego, CA.
