# Supplementary material for: Retrospective Study on Liberation in Dogs Undergoing Mechanical Ventilation for Longer Than 24 Hours
Source: J Vet Emerg Crit Care (San Antonio). 2026 Apr 18;36(2):235–47. doi: 10.1111/vec.70090 (PMC13150396; doi:10.1111/vec.70090)
Supplement: Supplementary file 1 — Supporting File 1: vec70090‐sup‐0001‐SupMat.docx. [file VEC-36-235-s001.docx]

| Patient no. | Ventilator mode at initiation of liberation | Duration of liberation (hours) | Time before liberation (hours) | PEEP (cm H_2_O) | Tidal volume (ml/kg) | PIP (cm H_2_O) | Set RR (bpm) | Time from liberation to extubation (hours) | Sedatives at time of liberation |
| --- | --- | --- | --- | --- | --- | --- | --- | --- | --- |
| 1 | AC - VC | 1 | **1** | 4.5 | 6.4 | 16 | 40 | 1 (tracheostomy tube removed) | Fentanyl 0.2 mcg/kg/min  Medetomidine 1.25 mcg/kg/hr  Midazolam 0.5 mg/kg/hr  Propofol 0.125 mg/kg/min |
|  |  |  | **0** | 4 | 11.7 | 13 | 28 |  |  |
| 2 | SIMV - PC | 2 | **2** | 3 | 9.8 | 9 | 28 | Tracheostomy tube in situ | Acepromazine 5mcg/kg  Medetomidine 0.5 mcg/kg/hr  Midazolam 0.2 mg/kg bolus after stopping CRI  Propofol 0.05 mg/kg/min  Trazodone 50mg (O-tube) |
|  |  |  | **0** | 2 | 7.9 | 8 | 28 |  |  |
| 3 | AC - VC | 1 | **1** | 3 | 8.4 | 11 | 36 | 0 | Ketamine 0.4 mg/kg/hr  Morphine 0.2 mg/kg/hr  Propofol 0.1 mg/kg/min |
|  |  |  | **0** | 3 | 7.3 | 11 | 36 |  |  |
| 4 | AC - PC | 1 | **1** | 5 | 5.4 | 8 | 20 | 0 (tracheostomy tube removed) | Sedatives not available |
|  |  |  | **0** | 5 | 8.5 | 7 | 20 |  |  |
| 5 | AC - VC | 2 | **1** | 5 | 6.7 | 21 | 18 | 1 | Acepromazine 5mcg/kg  Fentanyl 0.03 mcg/kg/min  Medetomidine 2 mcg/kg/hr  Midazolam 0.1 mg/kg/hr  Propofol 0.1 mg/kg/min |
|  |  |  | **0** | 5 | 6.4 | 20 | 18 |  |  |
| 6 | AC - PC | 1 | **1** | 4.5 | 5.7 | 19 | 40 | 1 (tracheostomy tube removed) | Fentanyl 0.5 mcg/kg/min  Midazolam 0.3 mg/kg/hr  Propofol 0.15 mg/kg/min |
|  |  |  | **0** | 4.5 | 6.3 | 13 | 40 |  |  |
| 7 | AC - PC | 1 | **1** | 4 | 5.8 | 9 | 20 | Tracheostomy tube in situ | Acepromazine 5mcg/kg |
|  |  |  | **0** | 4 | 7.8 | 15 | 20 |  |  |
| 8 | AC - VC | 0.75 | **1** | 3 | 9.5 | 19 | 15 | 0 | Fentanyl 0.03 mcg/kg/min  Midazolam 0.3 mg/kg/hr  Propofol 0.2 mg/kg/min |
|  |  |  | **0** | 3 | 6.7 | 13 | 6 |  |  |
| 9 | CSV -P | 2 | **1** | 4 | 7.9 | 7 | - | 0.5 | Sedatives not available |
|  |  |  | **0** | 3 | 13.5 | 7 | - |  |  |
| 10 | AC - VC | 1.5 | **1** | 5 | 9.2 | 13 | 37 | 2 | Fentanyl 0.03 mcg/kg/min  Midazolam 0.2 mg/kg/hr  Propofol 0.05 mg/kg/min |
|  |  |  | **0** | 5 | 12.8 | 21 | 37 |  |  |
| 11 | AC - VC | 1 | **1** | 5 | 8.8 | 19 | 30 | 2 | Fentanyl 0.2 mcg/kg/min  Midazolam 0.3 mg/kg/hr  Propofol 0.2 mg/kg/min |
|  |  |  | **0** | 5 | 8.2 | 17 | 45 |  |  |
| 12 | AC - PC | 1 | **1** | 3 | 7.7 | 13 | 30 | Tracheostomy tube in situ | Fentanyl 0.05 mcg/kg/min  Midazolam 0.3 mg/kg/hr  Propofol 0.3 mg/kg/min |
|  |  |  | **0** | 3 | 9.3 | 15 | 30 |  |  |
| 13 | AC - PC | 2 | **1** | 2.5 | 7.1 | 12 | 22 | Tracheostomy tube in situ | Fentanyl 0.2 mcg/kg/min  Midazolam 0.3 mg/kg/hr  Propofol 0.2 mg/kg/min |
|  |  |  | **0** | 2.5 | 8 | 11 | 22 |  |  |
| 14 | SIMV - PC | 1 | **1** | 5 | 11 | 18 | 35 | 0 | Dexmedetomidine 0.5 mcg/kg/hr  Fentanyl 0.2 mcg/kg/min  Midazolam 0.1mg/kg/hr  Propofol 0.1 mg/kg/min |
|  |  |  | **0** | 5 | 11.6 | 19 | 40 |  |  |
| 15 | AC - VC | 1.5 | **1** | 3 | 9.8 | 14 | 28 | 1.5 | Fentanyl 0.02 mcg/kg/min  Midazolam 0.2 mcg/kg/hr  Propofol 0.1 mg/kg/min |
|  |  |  | **0** | 3 | 8.5 | 13 | 22 |  |  |
| 16 | AC - PC | 2 | **1** | 1 | 6.5 | 11 | 35 | 2 | Fentanyl 0.1 mcg/kg/min  Midazolam 0.1 mg/kg/hr  Propofol 0.1 mg/kg/min |
|  |  |  | **0** | 1 | 9 | 11 | 20 |  |  |
| 17 | SIMV - PC | 1 | **1** | 4.5 | 10 | 21 | 37 | 1.75 | Fentanyl 0.06 mcg/kg/min  Midazolam 0.17 mg/kg/hr  Propofol 0.07 mg/kg/min  Acepromazine bolus (No dose recorded) |
|  |  |  | **0** | 4 | 9.1 | 21 | 37 |  |  |
| 18 | SIMV - PC | 3 | **1** | 5 | 5.6 | 15 | 30 | 0 | Butorphanol 0.2 mg/kg/hr  Fentanyl 0.2 mcg/kg/min  Midazolam 0.3 mg/kg/hr  Propofol 0.1 mg/kg/min |
|  |  |  | **0** | 5 | 7.8 | 16 | 30 |  |  |
| 19 | AC - PC | 0.5 | **1** | 4 | 9.8 | 21 | 40 | 0 | Fentanyl 0.05 mcg/kg/min  Midazolam 0.3 mg/kg/hr  Propofol 0.2 mg/kg/min |
|  |  |  | **0** | 4 | 10.7 | 22 | 40 |  |  |
| 20 | SIMV - VC | 3 | **1** | 2 | 8.7 | 20 | 20 | 1 | Fentanyl 0.08 mcg/kg/min  Propofol 0.2 mg/kg/min |
|  |  |  | **0** | 2 | 8.9 | 17 | 20 |  |  |
| 21 | AC - VC | 1 | **1** | 3 | 10.5 | 8.7 | 24 | 0 | Sedatives not available |
|  |  |  | **0** | 3 | 12.6 | 10 | 38 |  |  |
| 22 | SIMV - VC | 1 | **1** | 4 | 9 | 13 | 35 | 1 | Fentanyl 0.08 mcg/kg/min  Midazolam 0.2 mg/kg/hr  Propofol 0.05mg/kg/min |
|  |  |  | **0** | 3 | 9.2 | 7 | 20 |  |  |
| 23 | SIMV - VC | 0.5 | **1** | 5 | 5.5 | 5.2 | 40 | 0 | Acepromazine 20 mcg/kg  Fentanyl 0.2 mcg/kg/min  Medetomidine 2mcg/kg/hr  Propofol 0.1 mg/kg/min |
|  |  |  | **0** | 2 | 5.5 | 5.5 | 50 |  |  |
| 24 | SIMV – VC | 0.8 | **1** | 2 | 8.2 | 7.7 | 22 | Tracheostomy tube in situ | Acepromazine 2.5 mcg/kg  Fentanyl 0.08 mcg/kg/min  Propofol 0.15 mg/kg/min |
|  |  |  | **0** | 2 | 8.2 | 7.5 | 22 |  |  |
